# Everyday stress-induced neurobehavioral dysregulation: therapeutic targets for management with Neurexan

**DOI:** 10.3389/fpsyt.2025.1577689

**Published:** 2025-10-23

**Authors:** Stephan Duller, Gerard Clarke, Suzana Herculano-Houzel, Roland von Känel, Peter Kirsch, Hans-Peter Landolt, Britta Naschold, Stefan O. Reber, Rudy Schreiber, Martin Walter, George M. Slavich

**Affiliations:** ^1^ Elementrial Clinical Research, Graz, Austria; ^2^ Department of Psychiatry and Neurobehavioral Science and APC Microbiome Ireland, University College Cork, Cork, Ireland; ^3^ Department of Psychology, Vanderbilt University, Nashville, TN, United States; ^4^ Department of Consultation-Liaison Psychiatry and Psychosomatic Medicine, University Hospital Zurich, University of Zurich, Zurich, Switzerland; ^5^ Central Institute of Mental Health, Mannheim, Germany; ^6^ Institute of Pharmacology and Toxicology, University of Zurich, Zurich, Switzerland; ^7^ Heel GmbH, Baden-Baden, Germany; ^8^ Laboratory for Molecular Psychosomatics, Department of Psychosomatic Medicine and Psychotherapy, Ulm University Medical Center, Ulm, Germany; ^9^ Department of Psychopharmacology and Neuropsychopharmacology, Faculty of Psychology and Neuroscience, Maastricht University, Maastricht, Netherlands; ^10^ Department of Psychiatry and Psychotherapy at University Hospital Jena, Jena, Germany; ^11^ Department of Psychiatry and Biobehavioral Sciences, University of California, Los Angeles, Los Angeles, CA, United States

**Keywords:** life stress, RDoC, negative valence, pharmacological intervention, treatment

## Abstract

Everyday stress is highly prevalent and can impact human health, performance, well-being, and mortality risk. Despite being widely acknowledged, everyday stress is rarely assessed or treated, highlighting a critical gap in clinical routine. To better understand the relevance of everyday stress for healthcare professionals, we use the Research Domain Criteria (RDoC) framework to link stress neurobiology to its behavioral signs and symptoms. According to RDoC, everyday stress is conceptualized as impacting domains of basic neurobiological functioning. Everyday stress influences two primary domains – the negative valence domain, and arousal and regulatory systems – that can, in turn, lead to mental and physical health problems. Neurexan, a natural multi-component medication, targets these two domains. We review recent data on how Neurexan can mitigate the negative impact of stress on the negative valence domain and regulate arousal, thus helping to restore neurobiological and clinical functioning. This review provides an overview of relevant aspects of stress, mainly everyday stress, and the latest research on Neurexan as a potential option to target everyday stress. More broadly, this work may lead to new methods of assessing and managing everyday stress to improve the psychosocial resilience and well-being of individuals facing difficulty coping with repetitive or chronic stress in their daily lives.

## 1 Introduction

*Stress, in addition to being itself and the result of itself, is also the cause of itself*, was formulated as an uncomfortable paradox already in early times of stress research that addressed how multipronged and difficult to treat stress can be while not defining it (1). Similarly, while being an acknowledged risk factor for several physical and mental diseases, stress remains ambiguously defined, with conflicting interpretations and neglected health implications in terms of early detection, diagnosis, and management (2, 3). There has also been debate about ‘good’ vs. ‘bad’ stress and the blurred lines between the two when a normally beneficial stress response becomes maladaptive (4). The biological underpinnings of stress are equally ambiguous. Here, comprehending the intricate neurobiological processes, structures, and mechanisms of stress is essential for developing more effective treatments for preventing and reducing the negative consequences of stress and enhancing biopsychosocial resilience.

Despite these fundamental issues, stress is omnipresent in everyday life. Although stress can be both beneficial and harmful, as alluded to above, it is most used to refer to negative experiences that represent the daily grind of human life with significant health consequences. Indeed, the World Health Organization has identified stress as one of the most significant health challenges of the 21st century (5).

Although stress is not a disease itself, according to current classification systems such as the American Psychiatric Association’s Diagnostic and Statistical Manual of Mental Disorders (DSM) and the World Health Organization’s International Classification of Diseases (ICD), stress — or more accurately an individual’s biopsychosocial *response* to stressors — can be understood as a manifestation of neurobehavioral (dys)regulation within fundamental psychological and biological domains. In this context the National Institute of Mental Health (NIMH) introduced in 2009 the Research Domain Criteria (RDoC) initiative, which identifies fundamental brain functions with the aim of *transforming the understanding and treatment of mental illnesses.* The core principle of RDoC is rooted in the idea that research on mental illness should begin with the analysis of basic functions such as attention or response to threats (6), and consider that mental disorders represent disruptions in these functions. Here, we propose that conceptualizing stressor exposures — and the associated stress response and potential pathological consequences from the perspective of the RDoC framework — not only highlights everyday stress as a phenomenon of high clinical relevance but can help facilitate the testing of novel stress management strategies and treatments that may be useful for reducing stress and improving health.

Neurexan is one such possible treatment that we have studied. As a multicomponent medicinal product made from natural ingredients, Neurexan has been shown to affect multiple stress-related outcomes. To date, these effects have been demonstrated on molecular and physiological biomarkers, brain circuits, and patient-reported outcomes in a variety of controlled experiments (7–12). These effects on multiple stress biomarkers can be cross-referenced to various elements of the RDoC framework. This includes domains, such as negative valence, and constructs, such as acute threat (i.e., fear), as well as various units of analysis (or parameters), from neural circuits to self-reports of psychological phenomena.

In established nosological frameworks such as the DSM and ICD mental phenomena are predominantly characterized at symptom level. The present review does neither seek to replace these classifications nor to describe them as they are widely known.

The objective of this review is to complement the symptom-based approach by addressing neurobiological components in line with the above-mentioned RDoC framework established by NIMH. Firstly, the focus is placed on neurobiological mechanisms related to everyday stress and its correlates in the brain. Secondly, the evidence on Neurexan as a potential intervention for modulating everyday stress responses is emphasized using the RDoC framework.

## 2 Everyday stress affects health outcomes and mortality

In the fast pace of modern life, the perception of stress has become a constant companion. Stress is highly prevalent in the general population, with 62% of adults in California having encountered at least one stressful major life event during childhood (13). In the U.S., 85% of adults, and globally, 40% of adults, have reported feeling “stressed” in the past two weeks (13). Stress is implicated in nine of ten leading causes of death in developed countries and is the strongest predictor of all-cause mortality, surpassing smoking, obesity, and other causes (14). Moreover, the United Nations considers stress one of the most significant health problems of the 21st century (5), due to its contribution to numerous disorders such as depression and sleep problems (15), and several age-related diseases (16). All of these facts make stressor exposure, and the resulting maladaptive stress response, a well-established risk factor for poor health that affects multiple health-relevant systems such as the immune system, neuroendocrine system, autonomic nervous system, and microbiome, as well as human cognition, emotions, and behavior (17, 18).

### 2.1 Perception and appraisal of stress

In general, stress occurs in a situation where a stressor signals a threat to the organism (19). The stress response is a physiological reaction of the organism to deal with the threat. Stressors can be either physiological (e.g., heat, coldness, toxins) or psychological (i.e., new, uncontrollable, or unpredictable situations, or social evaluation). Moreover, stress can be classified based on its valence as good, negative, tolerable, or toxic stress; based on its duration as acute, repeated intermittent, or chronic; and based on the intensity, ranging from daily hassles to major life events (20).

Eustress, or good stress, is a type of stress that historically has been regarded as having a positive impact on an individual’s well-being, mental health, and behavior although responses can become maladaptive over time due to their effects on the sympathetic nervous system and hypothalamic-pituitary-adrenal (HPA) axis response (21, 22). Eustress is the opposite of distress, or negative stress that impairs functioning. The differentiation is not defined by the stressor, but rather by a person’s perception and appraisal of the situation influencing the stress response. The stress response in turn depends on several factors including one’s current feelings of control, desirability, location, and timing of the stressor. Tolerable stress refers to events of great adversity or threat, but individuals can manage and cope with these challenges (23). Coping is usually facilitated by the support of significant others, which strengthens the ability to regain a sense of control. Exposure to stressful and adverse experiences over a longer period without adequate coping can become toxic stress physiology that in turn increases the risk of developing health problems. Toxic stress physiology is an unmanageable stress response wherein previously health-promoting physiological dynamics are now harming health (e.g., through excessive inflammation and oxidative stress) (24).

Acute stress is a short-term, transient experience caused by a specific prompt, such as a traffic jam, an exam, or an argument with another person but also more severe prompts such as a natural disaster, a serious accident, or a sudden loss. The response to acute stress exposure typically subsides after the situation or circumstance has passed. If acute stress is experienced repeatedly over a long period of time, it can turn into chronic stress, which is experienced over months or years. Chronic stress can be ongoing or intermittent, and it can be caused by a variety of factors, such as a demanding work environment or a bad interpersonal relationship. Chronic stress can have a negative impact on mental and physical health, and lead to symptoms such as depression, anxiety, or high blood pressure (18, 25, 26). Repeated intermittent stress is a type of chronic stress that involves ongoing or frequently intermittent challenging life events that are associated with prolonged physiological changes, which may increase vulnerability to structural organ pathology.

Concerning the severity or intensity of stressors, one can differentiate between daily hassles and major life events. Major life events are more rare, significant, life-altering experiences such as marriage, divorce, birth, and death, whereas daily hassles are cumulative minor irritations and annoyances of everyday lives, such as traffic jams, lost keys, or arguments with friends or family (27). Daily hassles refer to the “irritating, frustrating, distressing demands that to some degree characterize everyday transactions with the environment” (28). What makes these occurrences a concrete hassle is the individual appraisal as such, rather than their mere occurrence (29). Despite the potential adverse effects of major life events on health, most individuals who encounter such events cope effectively and do not exhibit negative physical or mental health consequences (30). Cumulatively, daily hassles can exert a tremendous impact on mental health (28, 31–34). The cumulative-repetitive nature of the relatively minor life events is an even better predictor of the occurrence of subsequent clinical symptoms than major life event categories (34).

### 2.2 Cumulative stressors eroding resilience need early intervention

Adults consistently encounter numerous stressors throughout the day, leading to an accumulation of stressors as a common daily occurrence (35). This effect of cumulative stressors is notably more impactful than the influence of concurrent daily stressors (36). Stress becomes chronic when it persists for months to years. Chronic stress can in turn promote chronic low-grade inflammation (37, 38) and inhibit adaptive immunity, wound healing, biological repair mechanisms, and mental and physical performance (39). Chronic stress triggers both somatic and mental symptoms. Somatic symptoms include headache, chest pain, rapid heartbeat, and epigastric discomfort. Mental effects include nervousness, irritability, anxiety, tension, and feeling overwhelmed, besides cognitive symptoms such as fatigue, inability to focus, and confusion (18). Certain types of stressors may be particularly impactful, especially social stressors involving devaluation, rejection, exclusion, social isolation, living alone, and loneliness (14, 40, 41). In addition to the direct impact of chronic stress accumulation, daily hassles may lead to consuming comfort foods, excessive smoking or alcohol use, increased anxiety or depression, and disrupted sleep (42, 43). In the case of stress-induced disease pathogenesis, a series of steps can be formulated beginning with behavioral changes and ending with the development of a structural disease (44).

To better describe the situation when the environmental challenges exceed the individual ability to cope, the concept of allostatic load was introduced (45–47). This is the cumulative strain placed on mind and body in the form of both unrelenting stressors and stress responses. We emphasize the importance of recognizing that the same system that is needed for homeostasis in the face of stressors also leads to many of the common diseases of modern life when over-activated (48). Recently, there has been growing recognition within clinical practice of the importance of allostatic load in the balance between health and disease (49). To assess the allostatic load, integrated approaches including both biological markers and clinimetric criteria have been recommended (50). The measurements of allostatic load could enable clinicians to develop personalized interventions aimed at preventing or reducing the adverse effects of environmental factors on health (49).

Strategies for managing stress to reduce allostatic load may include various approaches, such as relaxation techniques, mindfulness meditation, yoga, psychotherapy, lifestyle modification, and medications (51, 52). A recent study from Denmark underscored that psychological stress is a prevalent cause of prolonged sick leave and a frequent reason for consulting a general practitioner (53). General practitioners’ management typically includes sick leave (54%), counseling (47%), pharmaceutical treatment (37%), and referrals to psychologists or psychotherapists (38%). However, to the best of our knowledge, there are currently no dedicated practice guidelines for the management of daily hassles available. General practitioners often have limited time per patient, leading to primarily symptomatic treatment of bodily symptoms rather than addressing the underlying neurobehavioral domains affected by stress. Public health care systems and psychotherapists typically address stress rather late, usually after significant progression in the accumulation of allostatic load, if they address it at all. Although interventions to alleviate stress and prevent disease manifestation may be reasonable at any stage of pathogenesis, early interventions hold the potential for the greatest beneficial impact on health and well-being.

Neurexan (Heel GmbH, Germany), a commercially available pharmacologic treatment, offers a promising early option for mitigating symptoms of everyday stress such as nervous restlessness (11) and associated sleep disturbances (12) before they escalate into more serious health concerns. Formulated with a defined combination of four diluted ingredients in measurable concentrations, Neurexan includes *Passiflora incarnata* (purple passionflower), *Avena sativa* (common oats), *Coffea arabica* (coffee plant), and *Zincum isovalerianicum* (valerianate of zinc) and acts as a multi-component, multi-target treatment designed to address stress through several mechanisms simultaneously. Importantly, it has been shown to be a safe option with a favorable tolerability profile and no known significant side effects, and its efficacy and safety have been investigated in several clinical trials and observational studies, supporting its role as an early and well-tolerated intervention in stress management.

### 2.3 Stress effects manifest in measurable neurobiological and systemic changes before subjective symptoms occur

Self-reported stress and symptom-based diagnoses, such as those outlined in the DSM-5 and ICD-11, rely on individuals’ subjective experiences of distress, mood changes, and functional impairment. However, subclinical stress symptoms also affect well-being and daily functioning even outside formal diagnoses. Research indicates that neurobiological and systemic changes often precede conscious awareness of stress or symptom manifestation, including rapid activation of the HPA axis, and autonomic nervous system (54). These early physiological responses mobilize energy and heighten vigilance, preparing the body to respond to perceived threats. Acute activation of the HPA axis results in cortisol release, modulating metabolism, immune function, and cardiovascular tone, while sympathetic activation increases heart rate, blood pressure, and catecholamine release, with concurrent suppression of parasympathetic activity. Over time, repeated or prolonged activation can contribute to dysregulation of metabolic, cardiovascular, and immune systems, linking stress physiology to long-term health outcomes (54, 55). A growing body of evidence links these systemic responses to the development and maintenance of stress-related symptoms, suggesting that objective biomarkers may complement subjective assessments in future diagnostic frameworks.

At the cellular level, stress fundamentally reshapes synaptic physiology through dynamic neural circuitry alterations. At the core of the neuroendocrine cascade, stress messages are transmitted via glutamatergic and GABAergic synapses within the hypothalamic paraventricular nucleus (PVN), regulating the initiation and termination of HPA axis activation and establishing adaptive feedback mechanisms (56). Beyond this, synaptic and structural plasticity in limbic and mesocorticolimbic systems relevant in mood and motivation are particularly vulnerable. In the hippocampus, chronic stress induces dendritic atrophy, reduced spine density, and suppressed adult neurogenesis, particularly in the dentate gyrus, contributing to cognitive deficits and impaired HPA axis feedback regulation (57, 58). Similarly, prolonged stress leads to dendritic spine loss and retraction in the prefrontal cortex (PFC) and altered spine density in the amygdala and nucleus accumbens, disrupting connectivity and contributing to maladaptive behaviors. More recently, research has highlighted that stress induces pathophysiological alterations at excitatory synapses, including disruptions in AMPA/NMDA receptor function, synaptic transmission dynamics, and neuroinflammatory modulation, as well as epigenetic regulation through DNA methylation, histone modifications, and non-coding RNAs that alter gene expression in stress-responsive neural circuits (59). These synaptic changes not only impair cognitive functions like working memory and emotional regulation but also establish a neurobiological substrate for long-term vulnerability to mood and anxiety disorders.

In parallel, the gut–brain axis has emerged as a critical pathway through which stress exerts systemic effects. The gut–brain axis represents a bidirectional communication system between the gastrointestinal tract and the central nervous system. It involves neural (e.g., the vagus nerve), endocrine, immune, and microbial pathways and plays an increasingly recognized role in how everyday stressors influence brain function and behavior. Landmark studies have shown that altering gut microbiota (e.g., through germ-free models or probiotic interventions) significantly impacts anxiety-like behavior, mood regulation, and neurotransmitter systems, particularly serotonergic pathways in the hippocampus (60, 61). Stress can disrupt the gut microbiome, leading to dysbiosis—a microbial imbalance associated with anxiety, depression, and other psychiatric conditions. Dysbiosis can trigger immune activation and systemic inflammation, which, in turn, modulate neural signaling and stress-related behavior, while gut microbes produce neurotransmitters such as serotonin and gamma-aminobutyric acid (GABA) that influence brain activity and emotional regulation. These findings paved the way to conceptualize the gut microbiome as a modulator of central nervous system activity. More recent reviews underscore that dietary and lifestyle factors shape gut microbial composition and function in ways that have downstream implications for cognition and emotional health, forming a “diet–microbiota–gut–brain” axis that offers promising targets for intervention in mood and stress-related disorders (62, 63). These converging lines of evidence illustrate that even minor daily stress can ripple through the gut–brain axis, altering immune signaling, neurotransmitter balance, and systemic inflammation, thereby shaping vulnerability or resilience to stress-induced psychopathology.

Neuroimaging research has provided significant insights into stress-induced brain changes and enriched our understanding of how everyday stress operates within the brain’s functional architecture. Early work using task-based fMRI revealed that exposure to psychosocial stressors robustly activates the amygdala, hippocampus, and orbitofrontal and medial prefrontal cortex, regions central to emotional regulation and stress processing (64, 65). Structural findings further indicate that chronic stress is associated with reduced hippocampal volume in stress-related disorders, underscoring long-term vulnerability (66). More recent longitudinal work demonstrates that acute stress-induced changes in large-scale networks, particularly the salience network (SN) and default mode network (DMN), predict long-term increases in perceived stress and post-traumatic symptoms; for instance, diminished connectivity between SN and DMN in response to stress heralded greater stress development over 16 months in a resilient cohort (67). Meta-analytical reviews indicate that acute stress triggers dynamic reconfiguration of large-scale brain networks, particularly through increased salience network engagement and decreased DMN and central executive network (CEN) connectivity, which suggests a reallocation of neural resources in the immediate stress response (68). A recent activation likelihood estimation (ALE) meta-analysis (69) showed that both physiological and psychosocial stressors consistently recruit the bilateral anterior insula and brainstem, reflecting shared salience and autonomic processes. However, physiological stress preferentially engaged the bilateral thalami, middle cingulate, and left fusiform gyrus, especially in men, while psychosocial stress elicited greater left amygdala activation, particularly in women, revealing stressor- and gender-specific neural signatures. Together, these imaging findings cast light on how everyday stress perturbs both structural and functional systems involved in emotion, attention, and regulation. These shifts may yield enduring impacts on mental health and resilience.

## 3 Stress and mental illness as dysfunctional neurobehavioral domains

Everyday stress is not merely a perceived phenomenon; rather, it is underpinned by intricate biological processes that can substantially affect health. To elucidate the biology of stress, we employed the research framework established by the NIMH’s RDoC project as a means to conceptualize the biosignatures associated with stress. RDoC, as a comprehensive research framework, facilitated our exploration of everyday stress as a manifestation of neurobehavioral dysregulation within fundamental neurobehavioral domains.

### 3.1 Symptom-based disease categories don’t support causal research

In the past, psychiatry has made little progress in translating biomedical research findings into clinical practice (70). Despite tremendous technological advances in basic scientific disciplines such as genomics and imaging, even the best treatments for mental disorders are effective in only about 50% of patients (71). One root cause is that the current classification systems of mental disorders are not biology-based. Rather, these classification systems, namely the American Psychiatric Association’s DSM and the World Health Organization’s ICD, apply a categorical approach based on self-reported or clinically observable symptoms neglecting the underlying biological pathologies of mental disorders. Several attributes of these symptom-based disease categories have been identified that hinder causal research for mental disorders (72). For instance, [1] disorder categories are not clearly separated from each other, with comorbidity being rather the rule than the exception; [2] different symptom profiles can lead to the same diagnosis; [3] assigning a mental diagnosis is a categorical yes/no decision that depends on partly arbitrary thresholds; and [4] symptoms overlap between different classes of disorders. The publishers of the DSM and ICD are aware of these limitations. The recently released ICD-11 made progress by considering symptom severity and time course, thus adding dimensionality; however, it still adhered to its symptom-based categorical approach. Perhaps as a response to the changes in the ICD, the Future of DSM working group is implementing similar changes (73).

One alternative diagnostic approach is to group patients based on neurobiological similarities, knowing that self-reported symptoms or observable psychopathology can differ significantly within the novel group definitions. However, this divergence could make it challenging for practitioners to diagnose patients based on clinical impressions and self-reports (74). It should also be emphasized that any modifications made to DSM or ICD criteria can cause significant disruptions across the mental health system affecting officially reported prevalence rates, insurance reimbursements, legal proceedings, disability declarations, and regulatory practices.

### 3.2 Adding neurobiological measures to the existing classification systems

To enhance psychiatric nosology and to create more biologically homogeneous subgroups, several approaches have been introduced in recent years. Examples are reverse nosology (75), Systems Neuroscience of Psychosis (SyNoPsis) (76), and Hierarchical Taxonomy of Psychopathology (HiTOP) (77). The RDoC project (78–82) has garnered the most significant attention among the novel approaches. The development of RDoC dates to Strategy 1.4 of the 2008 NIMH Strategic Plan: To develop, for research purposes, new ways of classifying mental disorders based on dimensions of observable behavior and neurobiological measures. It is crucial to understand that the RDoC is not meant as a substitute classification system but rather as a research framework that exists in parallel to the categorical systems. The purpose of the RDoC is to connect clinical observations to underlying neurobiology. It should be understood more like a process where concepts interact to link the understanding of genes, molecules, and cells to the intricate patterns of behavior and experiences in meaningful ways. The similarities between the RDoC and the DSM-5 are in their shared goal of enhancing the comprehension of mental disorders and their efforts to classify them (83).

The RDoC framework with the aim of transforming the understanding and treatment of mental illnesses was finally launched in 2009 and consists of a set of principles studying psychopathology based on dimensions of observable behavior and neurobiological measures. The concept is constantly evolving, as recently summarized (6).

Conceptually, the RDoC regards mental (psychiatric) illnesses as dysfunctions of brain circuits, whereas neurological and neurodegenerative disorders are typically characterized by localizable structural lesions. Techniques assessing brain activity, such as electroencephalography or functional neuroimaging, help pinpoint dysfunction in neural circuits. RDoC has several important characteristics. First, it integrates biosignatures from genetics, clinical neuroscience, psychology, environment, and development, thus enhancing the understanding of clinical symptoms and their management. Second, embracing a transdiagnostic approach, RDoC acknowledges that individual symptoms may manifest across various domains. For example, depression may be characterized by blunted neural function in positive and negative valence systems (84). Third, the framework employs a dimensional assessment that considers symptom severity as a continuum from normal to abnormal, rather than a strict yes or no categorization. This approach enables early-stage research into mental disorders, even in their subclinical phases.

### 3.3 RDoC Matrix to implement RDoC principles

The core principle of RDoC is rooted in the idea that research on mental illness should begin with the understanding of basic functions such as attention or response to threat (6). Disorders are seen as disruptions in these functions. Within RDoC, these functions are referred to as constructs, a term consistent with long-standing usage (85), and are categorized into overarching domains that encompass multiple related constructs. Constructs can be measured using different units of analysis, i.e. genes, molecules, cells, brain circuits, physiology, behaviors, self-reports, and paradigms. The RDoC matrix connects the domains with their constructs to the unit of analysis.

The current version of the RDoC matrix is constructed around six major domains of human functioning: negative valence systems, positive valence systems, cognitive systems, systems for social processes, arousal/regulatory systems, and sensorimotor systems (6). These domains reflect contemporary knowledge about major systems of emotion, cognition, motivation, and social behavior.

Negative valence systems are primarily responsible for reactions to negative, aversive situations or contexts, such as fear, anxiety, and loss. Positive valence systems are primarily responsible for reactions to positive, motivational situations or contexts, such as reward seeking, consummatory behavior, and reward/habit learning. Cognitive systems are responsible for various cognitive processes. Systems for social processes mediate responses in interpersonal settings of various types, including perception and interpretation of others’ actions. Arousal/regulatory systems are responsible for generating activation of neural systems as appropriate for various contexts and providing appropriate homeostatic regulation of such systems as energy balance and sleep. Finally, sensorimotor systems are primarily responsible for the control and execution of motor behaviors, and their refinement during learning and development.

Each domain contains several constructs and subconstructs. For example, within the negative valence domain, five constructs are differentiated: Acute threat (fear), potential threat (anxiety), sustained threat, loss, and frustrative nonreward. It is important to recognize that there is interaction and overlap among the constructs and domains. It is also important to note that the RDoC matrix is meant as a current snapshot of the principles (domains, constructs, units of analysis) envisioned to change over time. For example, the positive valence domain was significantly revised in 2016 and the sensorimotor domain was added only in 2019. Over time, the scientific community is encouraged to curate its own constructs (6, 78–80).

### 3.4 Everyday stress is related to domains such as *negative valence and arousal/regulatory systems*

The usefulness of the RDoC framework for research in various stress-related disorders has been widely acknowledged. Stress-related disorders frequently involve dysregulation across multiple domains. Negative valence systems exhibit heightened responses to aversive stimuli, increased fear, and sustained threat responses in stress-related disorders. Stress directly impacts arousal and regulatory systems, triggering physiological responses through the physiological effector system and the HPA axis, commonly dysregulated in such disorders. Cognitive processes, including attention, memory, and decision-making, are influenced by stress, often resulting in reduced cognitive performance. Stress-related disorders commonly manifest with cognitive deficits such as difficulties in concentration and memory retrieval (86). Chronic stress may also affect positive valence systems, leading to reduced interest or pleasure in rewarding activities, as reflected in symptoms like anhedonia. Additionally, stress can disrupt social functioning and interactions, with social support playing a crucial role in buffering stress’s impact. Disruptions in social processes are frequently associated with stress-related disorders. Core findings in neurobiological post-traumatic stress disorder (PTSD) research were matched to the RDoC research domains negative valence, cognition (87), positive valence, and arousal, the development of additional novel domains such as stress and emotional regulation and maintenance of consciousness (88) or traumatic stress spectrum, which spans both adaptive and adverse reactions to trauma (89) was suggested. Hence illustrating that the RDoC matrix is a flexible and evolving.

In adolescents, the consequences of chronic stress involve brain circuits, molecules, and behaviors that align with the construct of sustained threat within the negative valence domain of RDoC (90). In conclusion, markers of stress in general are found throughout all domains but are most often associated with negative valence, social processes, and arousal (91).

In relation to everyday stress, two constructs within the negative valence domain stick out: Acute threat (fear) and sustained threat are linked to acute and chronic stress, respectively. Acute threat is defined at the RDoC website (https://www.nimh.nih.gov/research/research-funded-by-nimh/rdoc) as the activation of the brain’s defensive motivational system to promote behaviors that protect the organism from perceived danger. Sustained threat is defined as an aversive emotional state caused by prolonged (i.e., weeks to months) exposure to internal and/or external condition(s), state(s), or stimuli that are adaptive to escape or avoid. The exposure may be actual or anticipated; the changes in affect, cognition, physiology, and behavior caused by sustained threat persist in the absence of the threat and can be differentiated from those changes evoked by acute threat. Further constructs of interest in relation to stress are potential threat (“anxiety”) within the domain negative valence and arousal within the domain arousal and regulatory systems. A shared feature between these stress-related constructs is HPA axis activity at the physiological (dysregulation of the axis) and molecular [Corticotropin-Releasing Hormone (CRF), Adrenocorticotropin Hormone (ACTH), cortisol] level. The involvement of the HPA axis in potential threat can be explained by the observation that already the anticipation of stress, without an actual stressful life event, triggers a stress reaction, like a sympathetic nervous system response that activates the immune system. The HPA axis is also a relevant system to induce arousal. Notably, a dysregulated HPA axis is given as a physiologic unit of analysis within the construct of sustained threat. The disruption of the HPA axis is hypothesized to lead to dysregulated stress response phenotypes, exacting a physiological cost on the organism commonly referred to as allostatic load (92). Increased HPA activity has been observed in anxiety, schizophrenia, and bipolar disorder, whereas decreased HPA activity has been observed in burnout, some forms of depression, and PTSD (93–95).

Interestingly, one of the behaviors linked to the construct of sustained threat is anxious arousal, thereby connecting sustained threat to the two constructs potential threat (anxiety) and arousal. Anxious arousal involves a range of symptoms associated with stress response such as increased heart rate, rapid breathing, restlessness, muscle tension, and a sense of impending danger or doom. The three constructs show significant overlap and interaction in relation to stress. Anxious arousal is a common feature of several stress-related mental disorders such as generalized anxiety disorder, panic disorder, PTSD, and certain phobias.

## 4 Neurexan affects stress-related domains of functioning

Neurexan is an early treatment option for mitigating symptoms associated with everyday stress. Two of its ingredients, *Passiflora incarnata* and *Avena sativa* have historically been employed for mild symptoms of mental stress and as sleep aids (96, 97). However, the potential synergistic effects of the combination remain uncertain. Notably, the chemical composition and mode of action of Neurexan have yet to be fully elucidated.

**Figure 1:**
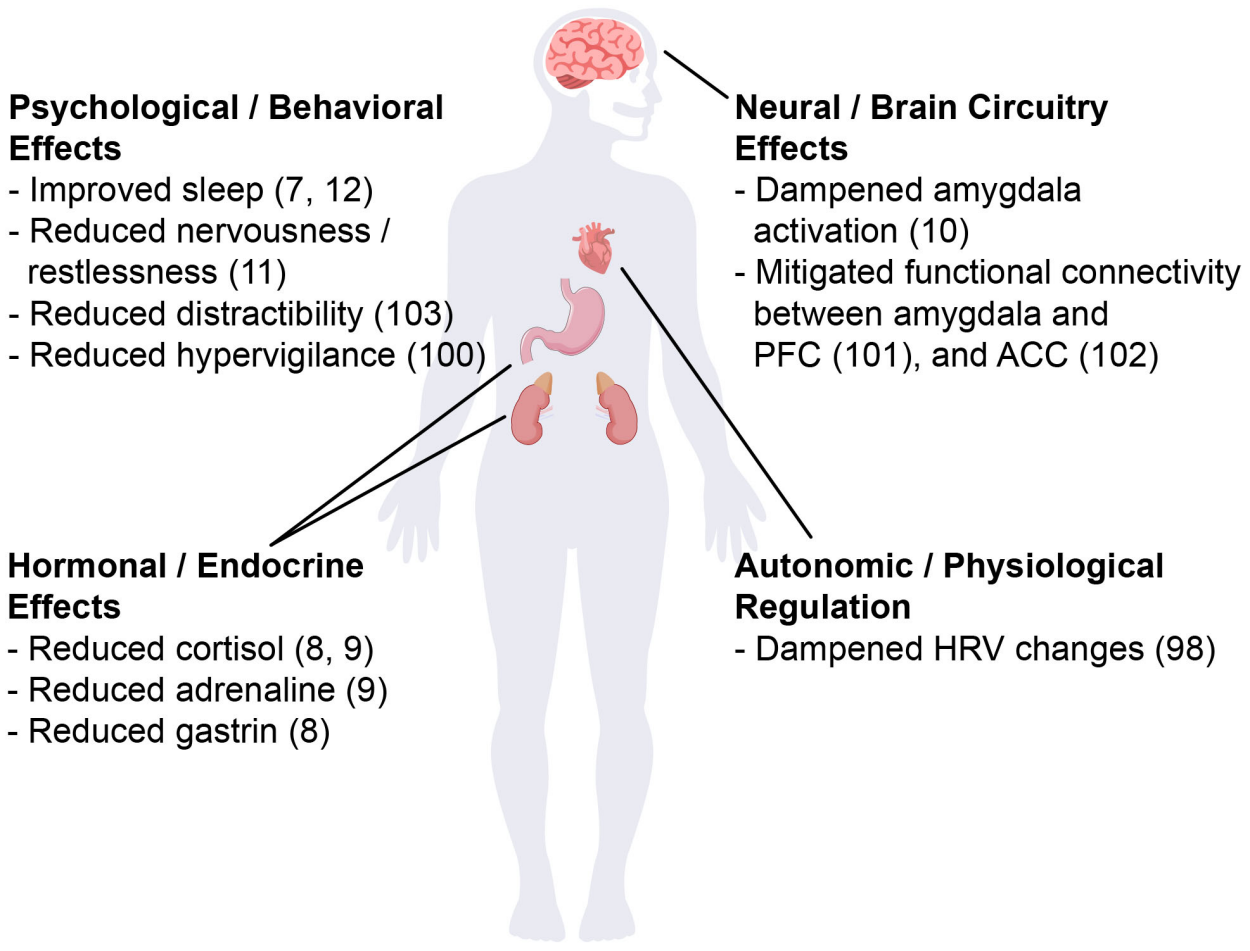
Schematic illustration of Neurexan’s effects on stress-related alterations. Neurexan was shown to improve sleep, reduce nervousness, distractibility, and hypervigilance, dampen amygdala activation and its connectivity to prefrontal cortex (PFC) and anterior cingulate cortex (ACC), lower cortisol, adrenaline and gastrin levels and stabilize heart rate variability (HRV).

Neurexan modulates stress responses through coordinated systemic and neuropharmacological mechanisms (**Figure 1**). Systemically, it dampens HPA axis hyperreactivity, reducing stress hormones such as cortisol and adrenaline as well as gastrin secretion (8, 9). Further Neurexan attenuates sympathetic activation, as reflected in heart rate variability changes, and mitigated stress-induced onset of insomnia (7, 98). These effects suggest that Neurexan dampens autonomic and gastrointestinal stress signaling, potentially reducing physiological arousal and improving sleep regulation. Neuropharmacologically, Neurexan modulates activity in limbic and prefrontal circuits central to stress and emotional regulation. Functional MRI studies demonstrate reduced amygdala reactivity and normalized amygdala–prefrontal connectivity, while EEG data show normalization of stress-related oscillatory activity and improved vigilance (10, 99–101). These effects suggest modulation of neural excitability and neurotransmitter systems, although precise molecular targets remain unclear. Strengths of this research include its multimodal approach, linking peripheral biomarkers, brain activity, and behavioral outcomes. Limitations include incomplete mechanistic understanding, and the predominance of acute stress paradigms, leaving chronic or real-world applicability uncertain. Overall, Neurexan demonstrates coordinated peripheral and central effects that mitigate physiological and neural responses to acute stress.

Several preclinical and clinical investigations have been conducted to enhance the understanding of Neurexan’s efficacy in treating nervous restlessness and related sleep disturbances (**Table 1**). To ensure the scientific quality of these studies, the manufacturer of Neurexan invited independent experts to an Expert Input Forum to discuss study design and analysis.

**Table 1:** Preclinical and clinical investigations to enhance the understanding of Neurexan’s efficacy in mitigating symptoms associated with everyday stress.

Species	Study type	Neurexan treatment (oral)	Stress symptom that was mitigated by Neurexan	RDoC construct	Study reference
Rat	Randomized, placebo-controlled, blinded	acute dose of ½ tablet per animal	Insomnia induced by male rat dirty cage exchange	Negative valence - acute threat	(7)
Dog	Randomized, placebo-controlled, blinded	acute dose of 10 tablets per animal over several hours	Elevated blood cortisol and gastrin levels in high-performance sled dogs during their intense training period	Negative valence - acute threat	(8)
Human (healthy)	Randomized, placebo-controlled, blinded	acute dose of 6 tablets over 2.5 h	Elevated salivary cortisol and blood adrenaline in response to stress task	Negative valence - acute threat	(9)
Human (mild to moderate chronic stress)	Randomized, placebo-controlled, blinded	acute dose of 3 tablets	Activated neural stress network in response to stress task	Negative valence - acute threat	(99)
			Resting state functional connectivity between amygdala and anterior cingulate cortex after stress task	Negative valence - acute threat	(102)
			Hyper-vigilance at resting state after stress task	Negative valence - acute threat	(100)
			Altered HRV after stress task	Negative valence - acute threat	(98)
			Activation of the amygdala in response to negative emotional task	Negative valence - acute threat	(10)
			Task-free resting state functional connectivity of prefrontal cortex and amygdala	Negative valence - acute threat	(101)
			Distractibility in an attention modulation task	Cognition - attention	(103)
Human (patients)	Non-interventional	at physician discretion over 2 weeks (recommended daily dose 3×1 tablets)	Nervousness/restlessness	Negative valence - sustained threat	(11)
Human (patients)	Non-interventional	at physician discretion over 4 weeks (recommended daily dose 3×1 tablets)	Insomnia	Arousal and regulatory systems - Sleep-Wakefulness	(12)

### 4.1 Neurexan mitigates acute stress-induced insomnia in rats

Neurexan’s impact on acute stress-induced onset insomnia was investigated in male Spraque Dawley rats after exposure to psychosocial stress factors, using the rat dirty cage exchange model (7). In this model (104), rats experienced stress upon being transferred to cages bearing scent markings of other males, which simulates territorial challenges. The transfer occurred just before their usual sleep time, triggering hyperarousal that resulted in stress-induced wakefulness or acute insomnia during the first hours after the cage exchange, mirroring acute stress-related onset insomnia in humans.

Rats were administered with Neurexan (approximately ½ tablet per animal) or vehicle, followed by a transfer to either dirty (stress) or clean (control) cages prior to their usual sleep time. Sleep variables, including sleep latency, wakefulness, rapid-eye-movement (REM) sleep, and non-REM sleep, were measured using surgically implanted EEG and EMG electrodes. Neurexan-treated rats exhibited reduced sleep latency after stress induction compared to the vehicle-treated group. Notably, sleep latency in Neurexan-treated animals did not significantly differ between clean and dirty-caged conditions. Additionally, in dirty cages, Neurexan-treated rats spent less time awake, had shorter wake episodes, and experienced more non-REM sleep compared to their vehicle-treated counterparts. This research indicates that Neurexan effectively alleviates or even prevents acute stress-induced sleep disturbance in rats. Given that the model is rooted in hyperarousal from the perception of an acute threat, Neurexan’s action aligns with alleviating effects on the negative valence domain of the RDoC system.

### 4.2 Neurexan reduced plasma cortisol and gastrin levels in sled dogs during an exercise-induced stress response

High-performance sled dogs undergo rigorous training to prepare for the demands of racing, with each session of practice creating a repetitive acute stress setting that elevates blood cortisol and gastrin levels (105). These stress biomarkers typically return to baseline within two hours post-training. Given the prevalence of gastritis and gastric ulcers in racing sled dogs, the heightened interest in gastrin, besides cortisol, stems from its role in stimulating gastric acid secretion (106), a pivotal factor in stress ulcer development.

Neurexan’s impact on cortisol and gastrin levels post-training was investigated in a randomized controlled animal study (8). As in the rat study described above, Neurexan was administered pre-exposure: Sled dogs received Neurexan or a placebo (10 tablets per animal) within three hours prior to engaging in a 35 km endurance race. Plasma cortisol and plasma gastrin were measured before and at various post-training time points. Results revealed a significant dampening effect on the exercise-induced increase in cortisol and gastrin in the Neurexan group compared to placebo. Given the similarity in cortisol response to both physical and psychological stressors through the HPA axis, these findings might have broader implications beyond the specific stressor type. As in the rat study, the observed mitigating effect on the HPA axis after acute stress setting also aligns with the acute threat construct in the *negative valence* domain of the RDoC system.

### 4.3 Neurexan diminished the increase of salivary cortisol in response to a stress task in healthy humans

Additional support for Neurexan’s stress-mitigating effects on cortisol release was obtained through a randomized controlled trial (RCT) in healthy humans (clinicaltrials.gov identifier: NCT01703819) that induced an acute stress response using the Trier social stress test (TSST) (9). The TSST is a widely utilized laboratory protocol simulating real-world stressors through a mock interview and mental arithmetic tasks in front of a panel of evaluative raters that aims to induce acute psychological stress and reliably elicit stress responses in both physiological and psychological domains (107).

In this clinical trial, participants challenged with the TSST experienced increased subjective stress ratings (Visual Analogue Scale for tension and nervousness), along with cardiovascular changes (increased blood pressure and heart rate) and alterations in neuroendocrine markers (release of cortisol in saliva, and of cortisol, adrenaline, noradrenaline, and ACTH in plasma). Notably, individuals administered with a single dose of Neurexan (i.e., 6 tablets over 2.5 hours) prior to stress induction exhibited significantly reduced stress-induced increases in salivary cortisol and plasma adrenaline compared to placebo. The TSST, as an acute psychological stressor, and the effects of Neurexan in this study align with RDoC construct acute threat in the *negative valence* domain. Importantly, these studies show that Neurexan is effective beyond placebo in curtailing acute stress responses in three mammalian species.

### 4.4 Results of the NEURIM trial: Neurexan reduced stress-induced hyperactivation in the brain and body

The neuronal correlates of Neurexan action were assessed in a comprehensive RCT (NEURIM trial; clinicaltrials.gov identifier: NCT02602275). This trial included participants experiencing mild-to-moderate chronic stress, who were exposed to various psychosocial stress paradigms, including an attention modulation task, the Hariri emotional face-matching task, and the ScanSTRESS paradigm which is an adaption of the TSST for use in functional Magnetic Resonance Imaging (fMRI) to induce psychosocial stress. The effects of a single dosage of 3 tablets of Neurexan administered prior to exposure to stress were compared to placebo using measures derived from EEG, fMRI, and Heart rate variability (HRV). In short, prior to experimental stress induction (101), Neurexan significantly decreased susceptibility to distraction in an attention task; modulated resting state functional connectivity associated with emotion regulation; and attenuated amygdala activation in response to negative emotional stimuli. During the experimental stress paradigm, there was a significant decrease in the activation of the anterior cingulate cortex as part of the stress network observed under Neurexan compared to placebo. Moreover, in the subsequent, post-stress resting state, Neurexan reduced the increased activity in the resting brain after psychosocial stress induction in individuals with high trait anxiety, improved stress biomarkers in EEG and HRV in a post-stress resting state, and enhanced vigilance regulation.

#### 4.4.1 Neurexan reduced the activation of the neural stress network in response to a stress task

In participants experiencing mild to moderate chronic stress, the ScanSTRESS paradigm (108) elicits a distinct pattern of activation in a stress network that comprises interconnected brain regions responsible for processing and regulating stress responses. Among the various affected brain regions identified during ScanSTRESS, Neurexan diminished activation of the supracallosal anterior cingulate cortex (ACC) (99). Specifically, the supracallosal segment of the ACC is known for its involvement in processing acute stress and negative valence emotions, such as punishment or unpleasant stimuli (109). These results further highlight the potential beneficial effect of Neurexan on the acute threat construct of the negative valence domain of RDoC.

#### 4.4.2 Neurexan dampened the post-stress resting state stress network activation in individuals with high trait anxiety

In the NEURIM trial, the activation of neural systems involved in stress as induced by the ScanSTRESS paradigm persisted into the post-stress resting state. Notably, individuals with high trait anxiety, a recognized vulnerability factor for stress-induced psychopathologies, exhibited a significant elevation in functional connectivity between the ACC and the amygdala during the post-stress resting state compared to the pre-stress condition. Neurexan administration prior to stress exposure attenuated the stress-induced alterations in resting state functional connectivity between these brain regions (102). Trait anxiety, functioning as a personality trait, along with the ScanStress paradigm, is intricately linked to the RDoC domain of negative valence systems.

#### 4.4.3 Neurexan improved vigilance regulation in the post-stress resting state

In addition to activating the neuronal stress network, the ScanStress paradigm also heightened the vigilance state of participants with mild-to-moderate chronic stress. EEG measurements revealed that, following the psychosocial stress task, the brains of participants remained in a hypervigilant resting state. Notably, under Neurexan treatment, participants experienced a significant reduction in stress-induced hypervigilance (100). The results are consistent with a stabilizing effect of Neurexan on the post-stress resting brain function, shifting it from a state of hypervigilance to one characterized by attentive calmness. Within the RDoC framework, hypervigilance, too, is commonly linked to the acute threat construct in the negative valence domain, representing an adaptive response to immediate threats. The observed effect of Neurexan in improving vigilance regulation further underscores its potential effect within the acute threat construct.

#### 4.4.4 Neurexan demonstrated correlated improvement of stress biomarkers in EEG and HRV in a post-stress resting state

A further variable affected by ScanSTRESS that persists well into the post-stress resting state is HRV. Stressor exposure causes a significant decrease in HRV between pre-stress and post-stress resting states, as indicated by reduced HRV parameter RMSSD (root mean square of successive RR interval differences), and the elevated HRV parameters Baevsky stress index, and LF/HF ratio. Treatment with Neurexan prior to stress exposure mitigated the stress-induced elevation of the stress index and LF/HF ratio. In parallel, stress led to altered EEG activity characterized by a decreased aperiodic offset, which was also diminished by Neurexan (98). Importantly, these two effects were correlated in magnitude which suggests a comprehensive intervention by Neurexan in buffering bodily responses to daily life stress. Given that the alterations in HRV and EEG are a consequence of the psychosocial stress induced by the ScanSTRESS paradigm, the effect of Neurexan on HRV in this context can also be aligned with the acute threat construct within the negative valence domain of RDoC.

#### 4.4.5 Neurexan reduced amygdala activation in response to negative emotional stimuli

Independent of the ScanSTRESS task, the NEURIM trial employed the Hariri Emotional Face Matching Task (110) to explore emotion regulation in mildly to moderately stressed participants. This task required participants to match faces expressing various emotional expressions (e.g., happy, fearful, angry) under different conditions. In the NEURIM trial, the Hariri face-matching task elicited amygdala activation in response to negative emotional stimuli. This finding is significant given the amygdala’s crucial role in efficient emotion processing and its sensitization under acute stress, leading to heightened anxiety. Notably, treatment with Neurexan prior to stress exposure had a mitigating effect on amygdala activation, particularly in the cortico-medial portion of the amygdala (10), a region of the structure that directly influences the physiological effector system and modulates the release of stress hormones (111). As emotional face-matching tasks involve stimuli depicting facial expressions of fear or threat, the observed responses can be linked to the acute threat construct within the negative valence domain of RDoC.

#### 4.4.6 Neurexan modulated task-free resting state functional connectivity indicating improved emotion regulation

The NEURIM trial also explored the impact of Neurexan on resting brain connectivity without any task or experimental stress induction. Using fMRI scans, global functional connectivity densities (gFCD) across various brain regions were assessed in the resting brain of participants reporting mild-to-moderate stress. Specifically, the medial prefrontal cortex (mPFC), recognized for its role in regulatory control of behavior during aversive events like stress or fear, was influenced by Neurexan: In the mPFC, gFCD strength significantly decreased after Neurexan treatment compared to placebo, consistent with an overall stress-relieving effect of Neurexan. Moreover, Neurexan was found to enhance functional coupling between the amygdala and prefrontal brain regions, which has previously been associated with more effective cognitive regulation of mood and anxiety. The association between these brain regions affected by Neurexan, and their connection to emotion regulation and stress response, further supports the alignment of Neurexan’s effects with the acute threat construct in the *negative valence* domain of RDoC.

#### 4.4.7 Neurexan reduced distractibility in an attention modulation task

An attention task based on the Attention Modulation by Salience Task (112, 113) was employed to examine the impact of Neurexan on susceptibility to distraction (103). During the task, participants were required to discern ascending from descending tones, while simultaneously being presented with images of varying salience (high/low) and valence (positive/negative) as distractors. Reaction time and event-related potentials (ERPs) recorded through EEG served as markers for evaluating susceptibility to distraction. In comparison to a placebo, prior treatment with Neurexan had significant effects, manifesting in quicker response times (improved reaction times) and reduced excitation, specifically dampening negative amplitudes N2 and N3. These effects were particularly pronounced when participants were exposed to positive emotional distractors. The findings suggest that Neurexan has a positive influence on attention that may stem from its ability to inhibit task-irrelevant information, thereby diminishing susceptibility to emotionally distracting stimuli.

The ability to suppress irrelevant information aligns with the attention construct in RDoC’s cognitive systems domain. While attention is also implicated in the negative valence domain’s sustained threat construct in the sense of attentional bias to threat, Neurexan’s greater impact on positive than on negative emotional distractors suggests a broader influence on general attention within the cognitive systems domain, in addition to the association with negative valence effects described previously.

### 4.5 Improved symptoms of nervousness observed under Neurexan intake

Neurexan exhibited effectiveness in treating symptoms of nervousness and restlessness, as demonstrated in a non-interventional study conducted in a primary care setting. This real-world study included 826 patients with clinical symptoms of nervousness and restlessness across 49 general practices in Germany (11). The participants were observed for two consecutive weeks while using Neurexan or Valerian-based products. The dosage was at the discretion of the physician. In the Neurexan group, almost 50% of participants received a daily dose of 3×1 tablets, and around 40% received >3 tablets a day.

The study assessed treatment effectiveness based on physician ratings in collaboration with the participants. Neurexan-treated individuals displayed positive outcomes across various variables, including nervousness/restlessness, excitability/jitteriness, sleep disturbances, hyperactivity, fragmented sleep, nocturnal anxiety, difficulties with concentration/forgetfulness, fatigue, listlessness, dysphoria, gastrointestinal disturbances, headache/pressure, and overall disease severity. Notably, Neurexan’s effectiveness surpassed that of Valerian products in improving participants’ perceived global state of nervousness/restlessness and overall health status. Nervousness, especially when it involves a persistent sense of unease or apprehension, may be indicative of an ongoing state of perceived threat or heightened arousal, aligning with the negative valence domain and sustained threat construct in the RDoC framework.

### 4.6 Improved insomnia symptoms observed under Neurexan intake 

Another non-interventional study followed 409 patients with mild-to-moderate sleep onset and/or sleep maintenance insomnia in 89 German general practices over 4 weeks (12). Patients who had been newly diagnosed or experienced recurring symptoms for ≤4 weeks prior to enrolment were treated with Neurexan or Valerian-based products. The dosage was at the discretion of the physician. All patients in the Neurexan group received the regular dose of one to three tablets per day. Sleep variables were collected in sleep diaries. After 4 weeks of treatment, both the Valerian and Neurexan groups reported improvements in sleep latencies, sleep duration, sleep quality, and daytime fatigue. Notably, both treatments reduced the number of patients on sick leave from employment due to insomnia.

Insomnia is related to the construct of sleep-wakefulness within the RDoC domain of arousal and regulatory systems. However, sleep can be influenced by negative valence, both in terms of sustained threat and acute threat. Chronic threat triggers prolonged release of cortisol, disturbing the sleep-wake cycle. Acute threat activates the locus coeruleus, which is arousing to brain and skeletal muscles, hindering relaxation and leading to insomnia or fragmented sleep. In both cases, the interplay between emotional processing and sleep regulation contributes to sleep disturbances.

## 5 Understanding stress on a neurobehavioral level and its improvement by Neurexan

Stress effects are observed across all domains of function, but the negative valence domain appears to be of paramount importance, particularly for acute effects of daily hassles that cumulatively lead to poor health. Neurexan is posited to exert its effects precisely at this initial stage. By ameliorating the impact of daily hassles at this critical juncture, we propose that Neurexan may short-circuit the downstream physiological stress response and thus help prevent the accumulation of allostatic load and mitigate negative effects on other domains of function. Although a broader effect of Neurexan on other domains is plausible, current evidence provides compelling support for Neurexan’s effectiveness in the negative valence domain. However, the multi-component nature of Neurexan makes a potential multitarget mode of action particularly likely, possibly spanning various domains of function. However, based on the current evidence within the framework, it is reasonable to assume that patients exhibiting deficits in the negative valence domain may benefit from Neurexan.

Based on the current literature, it is essential to acknowledge the limitations of our present understanding of Neurexan’s actions on the response to stress, which can in turn provide a useful guide for future research on this and other therapeutics. First and foremost, no specific studies have been conducted to investigate Neurexan’s effects on the remaining RDoC domains, which is a gap in understanding its comprehensive mode of action. Existing studies primarily focus on the effects of acute, experimentally-induced stress, rather than on patients experiencing chronic stress or clinically significant allostatic load. Studies on Neurexan’s effects on chronic stress or allostatic load are limited, with research comprising only two non-interventional studies focused on insomnia and nervous restlessness (11, 12). Additionally, all of the existing experimental studies on this topic have evaluated the effects of Neurexan through acute treatments prior to exposure to relatively short-term stressors, leaving the effectiveness of Neurexan for mitigating the negative impacts of long-term stressors unknown.

## 6 Understanding stress as a multidimensional phenomenon

Stress can be viewed as a complex interplay between major dimensions within the biopsychosocial model including not just biological factors but also psychological, and social domains (114). We thus do not view our approach as contradicting the biopsychosocial model, but rather as offering a complementary translational perspective within as we attempt to stitch together neurobiological factors and the RDoC model to make the case for a reliable framework to explain the role of stress management. Indeed, two of the four pillars of the RDoC framework – lifespan/development and environment - are actually not focused on biology. People often describe their symptoms in self-reports (e.g., tension, overwhelm, exhaustion), while neuroscience/biological psychiatry attempts to identify the biological causes (e.g., changes in neural circuits, cortisol release, altered gut-brain communication). These two perspectives do not always coincide - someone may report severe stress while their biological markers remain moderate, or vice versa - and there remains considerable debate regarding the evolution of the field of biological psychiatry (115). Differences arise from individual perception, coping styles, social context, and physiological sensitivity. This discrepancy underscores the importance of a comprehensive approach to the assessment, diagnosis, and treatment of stress and stress-related conditions. A top-down perspective (psychological and social interventions such as psychotherapy, coping skills, or mindfulness) addresses how people interpret and respond to stress. A neurobiological perspective (biological and physiological measurements such as cortisol, heart rate variability, or neurological treatments) captures the underlying biological processes. Combining both approaches leads to a more comprehensive picture and ultimately allows us to advance towards more effective, personalized care. For stress research, this means linking subjective experiences to measurable biological processes, considering genetic, molecular, and circuit-based mechanisms. Such an integrated approach offers the promise of a more precise holistic understanding and treatment of stress. The implementation of this framework should keep in mind important principles of the clinical management of stress-related disorders in relation to risk factors, the importance of addressing the underlying issues leading to impairments in stress coping and the appropriate deployment of psychological interventions instead of or in conjunction with psychopharmacological options.

## 7 Conclusion

Everyday stress can significantly impact daytime performance, multiple health outcomes, overall quality of life, and mortality risk through allostatic load. Despite its pervasive impact, however, everyday stress is rarely assessed or managed in clinical settings. This gap in addressing everyday stress underscores the pressing need for evidence-based strategies to alleviate its negative effects.

**Figure 2:**
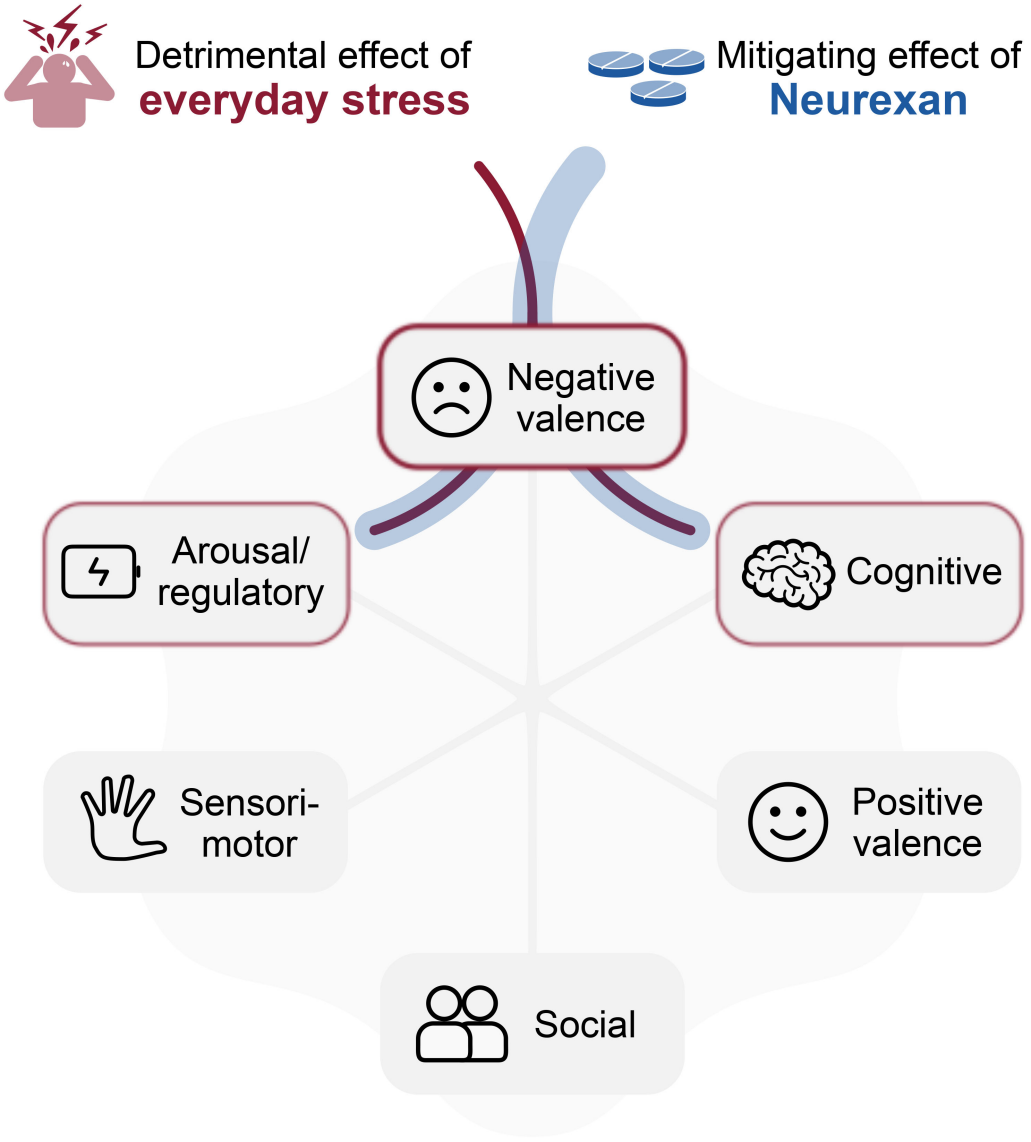
RDoC-informed model for anti-stress effect of Neurexan. Everyday stress predominantly impacts the negative valence domain within the RDoC framework and influences other domains due to their interconnected nature. Neurexan mitigates these detrimental effects, particularly targeting the negative valence domain. Additionally, Neurexan dampens arousal and improves regulatory balance, while supporting cognitive functions such as attention, thereby contributing to the overall restoration of system functionality.

The research framework RDoC has emerged as a valuable biopsychological framework for understanding the neurobehavioral dysfunctions associated with everyday stress across fundamental psychological and biological systems. By delineating major domains of brain function, the framework can help investigators explore the intricate interplay between stressors, the stress response, and various aspects of mental and physical health, and aging. Everyday stress primarily influences the *negative valence* domain, emphasizing the significance of aversive experiences and their psychological and neural mechanisms. However, the interconnectivity of the domains as the basis of the research framework becomes evident as chronic exposure to everyday stress manifests dysfunctions across multiple domains (**Figure 2**).

In this context, we described herein how Neurexan may be used as one potentially promising intervention for targeting RDoC domains negatively affected by stress. Existing data focus mainly on the negative valence domain; therefore, it remains unknown if Neurexan’s effects extend to other domains such as arousal and cognition, contributing to the restoration of overall system functionality. The potential for Neurexan to mitigate the negative effects of chronic stressor exposure also needs to be evaluated.

Ultimately, we believe that using the RDoC framework to study the potential benefits of Neurexan and other stress management strategies will help structure the field’s research approach and focus studies on mechanisms that may matter most for translating everyday stress into poor health. Importantly, such a framework should inform clinical management: recognizing risk factors, tailoring interventions to individual stress phenotypes, and determining when psychological interventions alone suffice or when pharmacological options like Neurexan may be indicated – also in conjunction with psychosocial care. This integrated, multidimensional approach promises a more precise and holistic understanding of stress and more effective strategies for prevention and treatment. Such an approach will help advance our understanding of mechanisms linking stress and health. More importantly, though, this approach will help us understand why the therapeutics we presently have work and what novel strategies may hold promise in the future.
